# Comorbid Chronic Pain and Posttraumatic Stress Disorder Among Veterans: Approaches to Care

**DOI:** 10.1093/milmed/usaf118

**Published:** 2025-04-11

**Authors:** Alessandra A Pratt, Jennifer Van Tiem, Brian C Lund, Nicole L Johnson, Kenda R S Steffensmeier, Daniel D Ball, Emily B K Thomas, Michelle A Mengeling, Sonya B Norman, Mary A Driscoll, Lauren Garvin, Kimberly J Hart, Katherine Hadlandsmyth

**Affiliations:** Center for Access & Delivery Research and Evaluation (CADRE), Iowa City VA Health Care System, Iowa City, IA 52246, United States; Center for Access & Delivery Research and Evaluation (CADRE), Iowa City VA Health Care System, Iowa City, IA 52246, United States; VA Office of Rural Health (ORH), Veterans Rural Health Resource Center-Iowa City (VRHRC-IC), Iowa City VA Health Care System, Iowa City, IA 52246, United States; Department of Family and Community Medicine, University of Iowa Carver College of Medicine, Iowa City, IA 52242, United States; Center for Access & Delivery Research and Evaluation (CADRE), Iowa City VA Health Care System, Iowa City, IA 52246, United States; VA Office of Rural Health (ORH), Veterans Rural Health Resource Center-Iowa City (VRHRC-IC), Iowa City VA Health Care System, Iowa City, IA 52246, United States; Center for Access & Delivery Research and Evaluation (CADRE), Iowa City VA Health Care System, Iowa City, IA 52246, United States; VA Office of Rural Health (ORH), Veterans Rural Health Resource Center-Iowa City (VRHRC-IC), Iowa City VA Health Care System, Iowa City, IA 52246, United States; Center for Access & Delivery Research and Evaluation (CADRE), Iowa City VA Health Care System, Iowa City, IA 52246, United States; VA Office of Rural Health (ORH), Veterans Rural Health Resource Center-Iowa City (VRHRC-IC), Iowa City VA Health Care System, Iowa City, IA 52246, United States; Center for Access & Delivery Research and Evaluation (CADRE), Iowa City VA Health Care System, Iowa City, IA 52246, United States; Center for Access & Delivery Research and Evaluation (CADRE), Iowa City VA Health Care System, Iowa City, IA 52246, United States; Department of Psychological and Brain Sciences, University of Iowa College of Liberal Arts and Sciences, Iowa City, IA 52242, United States; Center for Access & Delivery Research and Evaluation (CADRE), Iowa City VA Health Care System, Iowa City, IA 52246, United States; VA Office of Rural Health (ORH), Veterans Rural Health Resource Center-Iowa City (VRHRC-IC), Iowa City VA Health Care System, Iowa City, IA 52246, United States; Department of Internal Medicine, University of Iowa Carver College of Medicine, Iowa City, IA 52242, United States; National Center for PTSD, White River Junction, VT 05009, United States; Department of Psychiatry, University of California San Diego School of Medicine, La Jolla, CA 92093, United States; Pain Research, Informatics, Multimorbidities, and Education (PRIME) Center, VA Connecticut Healthcare System, West Haven, CT 06516, United States; Department of Psychiatry, Yale School of Medicine, New Haven, CT 06511, United States; Department of Psychiatry, University of Iowa Carver College of Medicine, Iowa City, IA 52242, United States; Department of Psychiatry, University of Iowa Carver College of Medicine, Iowa City, IA 52242, United States; Center for Access & Delivery Research and Evaluation (CADRE), Iowa City VA Health Care System, Iowa City, IA 52246, United States; VA Office of Rural Health (ORH), Veterans Rural Health Resource Center-Iowa City (VRHRC-IC), Iowa City VA Health Care System, Iowa City, IA 52246, United States; Department of Anesthesia, University of Iowa Carver College of Medicine, Iowa City, IA 52242, United States

## Abstract

**Introduction:**

The aim is to elucidate approaches to care for comorbid chronic pain and PTSD (CP + PTSD) in the Veterans Administration (VA). These conditions are co-magnifying and highly comorbid but traditionally treated in separate clinical settings.

**Materials and Methods:**

This multimethod analysis examined care for CP + PTSD via administrative data analyses and qualitative interviews of VA-served veterans.

**Results:**

All participants with diagnoses of CP + PTSD in 2021 were identified using VA administrative data (*N* = 456,544). Visits during the following year (2022) coded for chronic pain, PTSD, or both were analyzed. Qualitative interview participants (*N* = 22) were recruited, screened, consented, and enrolled in 2023. Administrative findings demonstrated that clinical settings differed where CP and PTSD were treated. For PTSD, 90.7% of visits occurred in the mental health service line, whereas for CP, visits occurred across a range of settings outside mental health (e.g., primary care, rehabilitative services, and surgical services). A small percentage of visits (4.8%) were coded for both CP + PTSD, indicating possible combined care. In qualitative interviews, participants acknowledged that CP and PTSD symptoms may impact one another but noted that the health care they received for these 2 conditions was typically siloed. Participants also identified barriers that would need to be addressed before a fully integrated coordinated care model could be implemented.

**Conclusions:**

Veterans reported interest in coordinated treatment for CP + PTSD; however, the provision of CP + PTSD care provided across different service lines may pose challenges to optimizing care coordination.

## INTRODUCTION

Chronic pain and PTSD (CP + PTSD) are prevalent and highly comorbid among U.S. military veterans.^1,2^ Among Veterans Administration (VA)-served veterans, over half (53%) of those with PTSD also have CP, and nearly one-quarter (22%) of those with CP also meet criteria for PTSD.^3^ Further, CP + PTSD can magnify the impact of one another; those with both conditions experience more intense pain, greater functional impairment, increased distress, poorer coping strategies, and higher healthcare utilization, relative to those with only one of these conditions.^4–8^

The mutual maintenance model of CP + PTSD outlines multiple ways in which trauma-related symptoms and pain can magnify and maintain one another.^9^ An example of overlying, co-magnifying symptomatology is the mutually reinforcing symptoms of hypervigilance and physiological reactivity (in PTSD) combined with interoceptive sensitivity and fear of physiological sensations (in CP), which can serve to both increase distress and impair physical functioning and quality of life.^9–11^

While the experiences of CP + PTSD may be inextricably linked for the individual patient, healthcare systems are typically designed to treat them as separate entities. A parallel treatment model, where 2 conditions are being treated simultaneously by different providers or teams, is common in the VA, particularly for the pharmacological management of CP + PTSD.^12^ Parallel care can be coordinated or siloed. If care is poorly coordinated, patients may become confused or overwhelmed, particularly if providers have conflicting treatment philosophies.^13,14^ A combined treatment model may mitigate risks associated with poor care coordination because one provider or team is responsible for treating CP + PTSD simultaneously. However, this model may be logistically challenging, particularly within a healthcare system where CP + PTSD are treated under different service lines.^15^

Current approaches to treating comorbid CP + PTSD in the VA need to be clarified. While combined care is increasingly being suggested as optimal in this population,^16–18^ additional detail is needed regarding current models of care, patients’ preferences for care, and potential barriers to optimal care. The current multi-method analysis sought to (1) identify settings in which CP + PTSD care is provided for veterans with both of these conditions via administrative data analysis and (2) qualitatively explore veterans’ experiences of care for CP + PTSD among veterans with both conditions.

## METHODS

### Administrative Data Methods

#### Data sources

Data were obtained from the VA Corporate Data Warehouse. Inpatient and outpatient visit data were used to build the cohort and identify visits for CP and/or PTSD. The administrative data analysis was determined nonhuman subjects research by the local Institutional Review Board.

#### Cohort

All veterans with diagnoses of CP + PTSD in their medical records in 2021 were included in the cohort. Veterans with PTSD were identified using the ICD-10 code (F43.1X) with at least one inpatient hospitalization coded for PTSD, or at least one PTSD-coded outpatient visit during 2021 and a second within the past 730 days.^19,20^ CP was identified using Tian’s criteria,^21^ modified to include ICD-10 codes.^22^ To meet criteria for CP, patients were required to meet one of the following 3 criteria: 2 outpatient visits separated by ≥30 days with a diagnosis code likely indicating CP^22^; at least 1 visit coded with a diagnosis likely indicating CP and at least 2 numeric pain rating scales ≥4; or long-term opioid use (>90 days).^19,23^ The cohort was built based on veterans meeting criteria for both CP and PTSD in 2021, and then the following year, 2022, was examined for the count of visits.

#### Analysis

Demographic variables assessed in the quantitative analysis were age, sex, race, and ethnicity. Patient residence (rural vs. urban) was also assessed using rural–urban commuting area (RUCA) codes.^24^ RUCA codes classified as urban were 1.0, 1.1, 2.0, 2.1, 3.0, 4.1, 5.1, 7.1, 8.1, and 10.1, while all others were classified as rural.^25^

The first analysis examined all outpatient workload visits (excluding labs) for the cohort and determined frequencies of visits coded for CP, PTSD, or both, and whether PTSD, CP, or neither, were the primary diagnosis for the visit (**Supplementary Table S1**). All visits that were coded with a primary diagnosis of CP or PTSD were examined for clinic type. These were then divided into visits where CP was the primary diagnosis and visits where PTSD was the primary diagnosis. The second analysis examined combined visits. A combined visit for CP + PTSD was defined as any visit during 2022 that was coded for both CP^22^ and PTSD (ICD-10 code F43.1X) with either CP or PTSD as the primary diagnosis for the visit.

### Qualitative Methods

The qualitative analyses presented in this article were part of a larger needs assessment project that documented the experiences of rural-dwelling veterans with CP + PTSD seeking care in the VA. This specific subanalysis aimed to characterize veterans’ health care for CP + PTSD and their care preferences.

#### Participants and procedures

**Figure 1.**
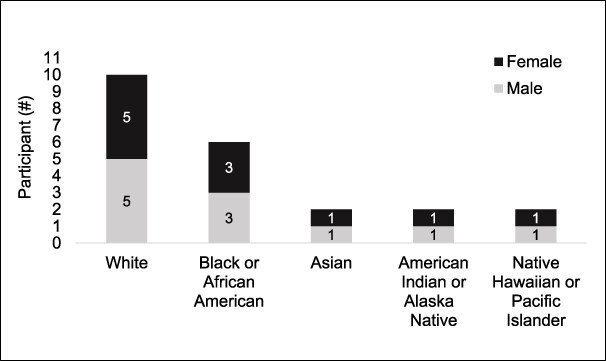
Qualitative interview participant demographics (*N* = 22).

This study interviewed rural-dwelling veterans with both chronic pain and PTSD until data saturation was reached, which was expected to be after about 20 interviews. Potential participants were selectively sampled from the study population to include black, indigenous, and other veterans of color and an equal number of women and men (Figure 1). Inclusion criteria were (1) VA-served veterans, (2) diagnosed CP, (3) diagnosed PTSD, (4) currently rural-dwelling, (5) access to a telephone, and (6) absence of any language or sensory impairment precluding verbal communication in English. Potential participants were selected via administrative data based on a rural address (determined by RUCA codes), ≥2 instances on separate visits of diagnoses likely indicating CP in the past year,^22^ ≥2 numeric rating scale scores indicating moderate to severe pain (≥4), and ≥2 instances on separate visits of diagnosis indicating PTSD (ICD-10 code F43.1x). The order in which potential participants were contacted was randomly assigned by an algorithm written by the data manager and stratified to oversample women and veterans from minoritized racial groups. Participants were randomly selected to be contacted and additional veterans continued to be contacted and offered interviews until data saturation was reached.

A research team member sent letters and followed up with phone calls about the study to each of the randomly selected potential participants. Those who were interested were verbally screened and verbally consented. Rurality, CP, and PTSD were verbally confirmed with each participant during screening. Demographic and treatment data were collected verbally, and a qualitative interview was scheduled. Letters were sent until data saturation was met via qualitative interviews.

A total of 72 potential participants were contacted. Eighteen participants declined to participate, 27 were unreachable, 1 was excluded (due to the participant reporting no diagnosis of PTSD on verbal screening), and 26 veterans verbally consented to participate. Among the 26 veterans who verbally consented to participate, 22 completed a qualitative interview, and 4 were lost to follow-up. Participant ages ranged from 28 to 74 years (*M* = 51). The study included veterans from the Navy, Air Force, Marine Corps, and Army. Participants resided in 19 different states. Eight participants were employed at the time of the study, 9 were retired, and 5 were either unemployed or disabled. In nonmutually exclusive categories, 19 participants reported currently seeking health care at a VA medical center, 14 participants reported currently seeking health care at a community-based outpatient clinic, and 15 participants reported currently seeking health care via the VA’s Community Care program.^26^ VA’s Community Care program provides care to veterans via community providers and is based on specific eligibility requirements and availability of VA care.^26^

#### Data collection

Four qualitative analysts trained in anthropology, public health, or health communication conducted semi-structured interviews that lasted an average duration of 62 minutes (range 33-104). Interview questions elicited data about participants’ experiences living with CP + PTSD, treatments received, the potential utility of treating CP + PTSD together, communication with their healthcare providers, and their experiences with medications for these conditions. This article reports on participants’ perceptions of the potential utility of treating CP + PTSD together. The study was approved by the local Institutional Review Board.

#### Data analysis

Interviews were audio recorded and auto-transcribed, and the qualitative analysts checked each transcript for accuracy. The qualitative analysts used MAXQDA, a qualitative data management and analysis software, to code each interview.^27^ Two qualitative analysts coded the transcripts using deductive codes derived from the interview guide, as well as inductive codes, which were identified and discussed as patterns developed. Both analysts coded all the interviews together; for the first 3, they met and coded together; and for the remaining transcripts, they coded separately and then checked each other’s work, meeting to discuss discrepancies and emerging patterns.

This article reports on a thematic analysis^28^ of 2 codes (barriers to care and combined treatment intervention) by 2 of the coauthors (J.V.T. and A.A.P.). Pile sorting was used to organize segments with similar meanings. Patterns within and across groups were noticed as groups were categorized. Example categories were CP + PTSD interaction, physician communication and changing providers, VA being difficult to navigate, and considerations for combined treatment. Microsoft Excel was used to further identify cross-cutting patterns across groups. Three cross-cutting patterns that characterized participants’ preferences were identified, including experiences of uncoordinated care, perceptions of a mismatch between their care and their condition, and challenges navigating the VA healthcare system (see **Supplementary Figure S1** for the thematic map). Pseudonyms are reported to protect confidentiality.

## RESULTS

### Administrative Data Results

In 2021, a total of 456,544 veterans received care at the VA for CP + PTSD. One-third of the veterans were ≥65 and resided in a rural area (**Table 1**). The cohort was majority male (83.0%), white (64.9%), and not Hispanic or Latino/a (89.9%).

**Table 1.** Patient Characteristics of Veterans With CP + PTSD in 2021

Characteristic	*N* = 456,544, *n* (%)
Age, years	
<40	95,587 (20.9)
40–54	118,681 (26.0)
55–64	88,345 (19.4)
65+	153,931 (33.7)
Sex	
Male	379,060 (83.0)
Female	77,484 (17.0)
Patient residence	
Rural	156,392 (34.3)
Urban	300,152 (65.7)
Race	
White American	296,348 (64.9)
Black or African American	115,871 (25.4)
Hawaiian or other Pacific Islander	6,390 (1.4)
Asian American	6,801 (1.5)
American Indian or Alaska Native	6,684 (1.5)
Ethnicity	
Hispanic or Latino	46,145 (10.1)
Not Hispanic or Latino/a/x	410,399 (89.9)

Among all outpatient visits for CP or PTSD in 2022 (3,974,311), 90.7% of PTSD visits occurred in the mental health service line, compared to only 2.1% of CP visits occurring in the mental health service line (**Table 2**). When CP was the primary diagnosis, the greatest frequency of visits occurred in rehabilitation services (21.6%) or primary care (18.7%). Rehabilitation services was the second most common clinical service line when PTSD was the primary diagnosis, but only accounted for 2.5% of visits. Among all outpatient visits with a primary code for CP or PTSD, only 4.8% of visits were coded for both CP + PTSD (190,486 visits). This implies that a combined care model was utilized in up to 4.8% of visits.

**Table 2.** Distribution of Clinic Types for Visits in 2022 Where Either CP or PTSD Was the Primary Diagnosis Among Veterans Diagnosed With Both CP and PTSD

Clinical service	CP visits (*N* = 1,646,449), *n* (%)	PTSD visits (*N* = 2,327,862), *n* (%)
Mental health	35,217 (2.1)	2,113,183 (90.7)
Not mental health	1,611,232 (98.0)	214,679 (9.3)
Pain clinic	141,110 (8.6)	3,161 (0.1)
Primary care	307,209 (18.7)	51,576 (2.2)
Rehabilitation services	356,147 (21.6)	57,056 (2.5)
Other^a^	806,766 (49.1)	102,886 (4.5)

a Other included medical services includes neurology, surgery, and any other clinic types not specified.

### Qualitative Results

To understand veterans’ perspectives on the potential feasibility and utility of coordinated care for CP + PTSD, veterans were asked if it would be useful to treat their CP + PTSD symptoms together, and if so, what would be most helpful. Veterans were asked to describe their current care, including where they typically go and who they typically see, and to describe their preferences for a hypothetical combined intervention. Veterans described their perceptions that their PTSD and CP symptoms may be connected, but that the care they received does not reflect their experience of their condition and is often uncoordinated. In outlining their current care and their preference for care, they discussed the context of the VA as a healthcare system. Veterans’ reflections on their current healthcare experiences, as well as their preferences for ideal health care, clarified the ways in which they were trying to get care in a system that is not structured to optimally support coordinated care for these 2 interrelated chronic conditions.

#### Willingness to try coordinated treatment

Of the 22 people who were interviewed, most participants (*n* = 20) reported that they would be willing to try a coordinated intervention. Responses varied for the exact model of coordination. However, it was clear that veterans recognized that their CP + PTSD were interrelated and that they were willing to try some method of coordinated treatment. In describing why they were willing to try a coordinated treatment, veterans expressed frustration with uncoordinated, siloed care, and how siloed care did not match up with their experiences of their bodies. One person remarked:

If you can find one doc that can do both, I’d like to see that person … providers specialize, they don’t want to treat the whole person as a whole person … [One provider says] “well, I’m gonna treat your brain,” [and another provider says] “and I’m gonna treat your neck” … [but] it doesn’t work like that. It’d be great if it did, but it doesn’t. (Joanne)

Another person’s reflections expanded on this disconnection between their lived experiences with CP + PTSD and their experience of care for those conditions, and how siloed care impacted their lives more broadly, they explained how,

A lot of it goes together … if you’re not in a good mental space then your body is kind of following that situation and your pain can be … more prominent on those days. So I do think that it would help for them to be treated together. I think that if the doctors were working together they could find a medication that could help both situations … [but at the moment,] you’ve got one doctor going well you need to take this medication and it makes you sleepy and you got this medication over here that the doctor’s giving you for pain and it makes you even sleepier, [so now] you’re gonna be in the bed all day. (Diane)

Veterans also highlighted barriers which they believe need to be addressed to facilitate coordinated care. Veterans detailed 2 ways that their current care is siloed, and how their experiences trying to get care could exacerbate their symptoms. Veterans also reflected on how systemic issues became a barrier to maintaining care that worked for them.

#### Barrier 1: Siloed care exacerbates symptoms

Participants described 2 kinds of siloed care provision, including (1) seeing one doctor for one issue and another doctor for another issue and (2) having to switch providers often. Seeing one “doctor for my mental stuff, then one for pain” felt like “going around in circles”; this person thought that “if it was kind of more centralized … it would be a lot less stress and mental wear” (Mark). Where this participant described “stress” and “mental wear,” another participant described feeling “numb” after having to switch providers often. They said, “it’s a constant change … [and] I feel like every time I change, I have to start over … and at this point I’m just kind of numb to that” (Diane).

#### Barrier 2: Trying to get approvals for care can create confusion and frustration

In addition to uncoordinated care, participants also described the VA’s structure for approvals for specific types of treatment, particularly if it requires reimbursement to a community-based provider, and its accompanying frustration and confusion. One participant explained how:

The VA approves something that’s actually great, and then all of a sudden it gets taken away because, “Well we only allow it for X amount of weeks, and then you have to go back and try and get it authorized again.” (Andrew)

When Andrew was unable to get a subsequent authorization, having the care “suddenly taken away” was then compounded by an “ongoing battle” to continue to receive care that was “actually great.” They explained:

I didn’t realize that it would be an ongoing battle back and forth with the VA to authorize it like on a daily basis … it was just confusing and frustrating along those lines and I don’t know why … it seems like a battle to try and get it authorized once again. (Andrew)

Whether having to see multiple doctors, or switching doctors, or “battling” the system to get the care they needed, participants described not being able to make sense of their care. While the participants were willing to try a coordinated care approach, they seemed to also recognize that the utility of that approach would likely be limited by system-level barriers.

## DISCUSSION

Taken together, these findings indicated that veterans would like coordinated treatment for CP + PTSD; however, there are system-level barriers. From the veterans’ perspectives, they had many providers and medications that were individually managed. From an administrative data perspective, CP + PTSD were typically treated in different settings under different service lines, and there was a very limited indication of combined care.

Administrative data findings highlighted how the structure of health care can reinforce siloed care. Among this population of veterans with both conditions, the majority of PTSD treatment occurred in the mental health service line, while CP care was provided in a range of clinical settings and service lines (i.e., primary care and specialty care). Transitioning toward integrated models of coordinated care for CP + PTSD may require system-level support. Recent transitions within the VA toward a Whole Health approach to care,^29,30^ as well as implementation of primary care mental health integration clinics, and embedding behavioral health clinicians in CP management teams^31^ are examples of structural transformations that can help support coordinated and combined models of care. In addition, increasing use of case management care coordinators to coordinate care between chronic pain and PTSD providers may also enhance coordinated care for veterans. Further, leveraging telehealth may also prove critical in providing such care, particularly for veterans residing in rural and remote locations.

Qualitative findings indicated that veterans were interested in a coordinated care approach; however, they also perceived limitations in the VA healthcare system (e.g., switching providers, seeing many different specialists, and having to navigate bureaucratic rules for approvals for care in the community) as barriers to the implementation of a coordinated model. These findings are consistent with prior work identifying barriers to veteran-preferred care in the VA.^32–36^ Specifically, the results of this work mirror recent findings from 2 studies that identified barriers to veterans with PTSD receiving mental health care at the VA.^32,36^ Both studies found that veterans’ treatment preferences were not met, there were logistical issues with treatment access and coordination, and their past experiences with receiving treatment (e.g., staff turnover and inability to establish a consistent provider) acted as barriers to care. System-level barriers to continuity of care, such as the requirement for a series of referrals and authorizations between different service lines and specialties, have also been identified in other qualitative work.^37^ Future research is needed to further understand how a coordinated treatment model for CP + PTSD could be fully implemented within the VA.

This study has several limitations. Findings from within the VA healthcare system and qualitative interviews with rural veterans may not generalize to other disparate treatment settings. Another limitation is the constraints of looking at visit-level data to approximate combined care; future work using chart review is needed to further assess evidence of combined and coordinated care. Finally, future work is also needed to better elucidate provider coding behaviors and whether providers are fully capturing the extent of what is covered in their clinic visits in their coding practices.

In conclusion, our findings indicate that while veterans would like a coordinated treatment for CP + PTSD, there are system-level barriers to implementing this model of care. While the VA has worked to implement a more comprehensive model of care for the whole person, there remain structural barriers to address in order to fully implement coordinated care for CP + PTSD.

## Supplementary Material

usaf118_Supplementary_Data

## Data Availability

These data are not available on request to the authors; they belong to the Veterans Health Administration which can grant access.

## INSTITUTIONAL CLEARANCE

Institutional clearance approved.
