# Supplementary material for: Comorbid Chronic Pain and Posttraumatic Stress Disorder Among Veterans: Approaches to Care
Source: Mil Med. 2025 Apr 11;190(9-10):e2058–64. doi: 10.1093/milmed/usaf118 (PMC12596722; doi:10.1093/milmed/usaf118)

Supplemental Figure 1. *Thematic map of findings demonstrating the barriers to Veterans' willingness to try coordinated treatment for co-occurring PTSD and chronic pain.*

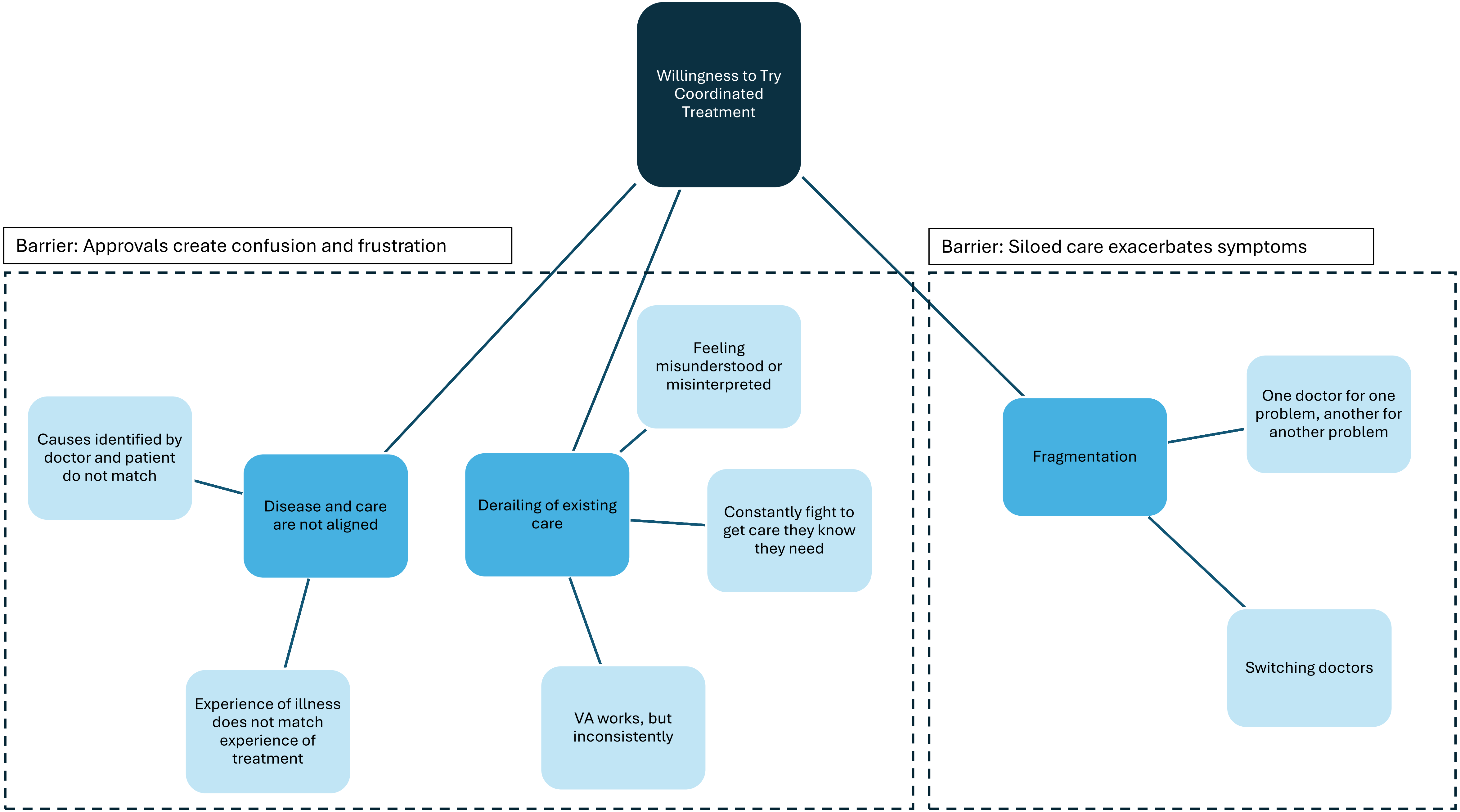

Supplement: usaf118_Supplementary_Data [file usaf118_supplementary_data.zip › Supplemental Figure 1_Thematic map_final.pdf]
