# Supplementary material for: Comorbid Chronic Pain and Posttraumatic Stress Disorder Among Veterans: Approaches to Care
Source: Mil Med. 2025 Apr 11;190(9-10):e2058–64. doi: 10.1093/milmed/usaf118 (PMC12596722; doi:10.1093/milmed/usaf118)
Supplement: usaf118_Supplementary_Data [file usaf118_supplementary_data.zip › Supplemental Table 1_Pain and PTSD care_12.16.24.docx]

Supplemental Table 1: Primary and secondary codes for CP or PTSD among outpatient Visits for patients with both CP+PTSD.

|  | Total visits  N = 5,755,405 | |
| --- | --- | --- |
| Visit codes | n | % |
| Both CP+PTSD coded | 410,746 | 7.1 |
| CP coded as primary | 108,041 | 1.9 |
| PTSD coded as primary | 82,445 | 1.4 |
| Both coded as secondary | 220,260 | 3.8 |
|  |  |  |
| Only CP coded | 2,107,332 | 36.6 |
| CP coded as primary | 1,538,408 | 26.7 |
| CP coded as secondary | 568,924 | 9.9 |
|  |  |  |
| Only PTSD coded | 3,237,327 | 56.2 |
| PTSD coded as primary | 2,245,417 | 39.0 |
| PTSD coded as secondary | 991,910 | 17.2 |
